# Bloom syndrome: research and data priorities for the development of precision medicine as identified by some affected families

**DOI:** 10.1101/mcs.a002816

**Published:** 2018-04

**Authors:** Mary Beth Campbell, Wesley C. Campbell, James Rogers, Natalie Rogers, Zachary Rogers, Anne Marie van den Hurk, Annie Webb, Talon Webb, Paul Zaslaw

**Affiliations:** 1 2017 Pediatric Cancer Nanocourse, Children's Cancer Therapy Development Institute, Beaverton, Oregon 97005, USA;; 2 Caltech Office of Technology Transfer and Corporate Partnerships, California Institute of Technology, Pasadena, California 91125, USA;; 3 Department of Physics & Astronomy, University of California Los Angeles, Los Angeles, California 90095, USA

## Abstract

Bloom syndrome (BS) is a rare, autosomal recessive genetic disorder characterized by short stature, a skin rash associated with sun exposure, and an elevated likelihood of developing cancers of essentially all types, beginning at an early age. Cancer is the leading cause of death for persons with BS, and its early onset results in a reported median lifespan of <30 years. With fewer than 300 documented cases since BS was first described in 1954, its rarity has challenged progress in advancing both the care of and the cure for persons with BS. Presently, there are no known clinically actionable targets specific to persons with this cancer predisposition syndrome, despite the fact that standard cancer treatments are often contraindicated or must be substantially modified for persons with BS. Herein, Zachary Rogers recounts his experience as a cancer patient with BS contemplating a substantially customized chemotherapy regimen that highlights the need for development of individualized treatments in the BS community. We also outline a patient-centered research and community action road map with the goal of improving and prolonging the lives of persons with Bloom syndrome, including the facilitation of precision medicine development specific to this condition.

## INTRODUCTION

In 1954, David Bloom, a dermatologist in New York City, reported the cases of three children with short stature and telangiectatic erythema, and noted that the cause was likely a genetic syndrome (Bloom 1954). Bloom syndrome (BS) is now known to be an autosomal recessive genetic disorder affecting the *BLM* gene, which codes for a DNA helicase protein in the RECQ family (Cunniff et al. 2017). Since BS was first reported, approximately 300 cases have been documented (Sanz et al. 2006). Since 1960, the Bloom's Syndrome Registry^4^ (BSR) has existed to collect biological samples and natural histories of persons with BS. As recently described in a review (Cunniff et al. 2017), BS is clinically variable and has primary features that include growth, dermatologic, and endocrine issues, along with a greatly increased risk of cancer. From a molecular perspective, the *BLM* gene is one of the so-called “guardians of the genome” because of its importance in maintaining the structure and integrity of DNA (Larsen and Hickson 2013); without the BLM protein, cells exhibit features of chromosomal instability, leading to the greatly increased incidence of cancer seen in BS. The molecular functions of BLM are many, including rescuing stalled forks and dissolving double Holliday junctions; and BLM interacts with other DNA-repair proteins including BRCA1, ATM, and RAD51. These functions and interactions are further described in Cunniff et al. (2017).

As part of the 2017 Pediatric Cancer Nanocourse hosted by the Children's Cancer Therapy Development Institute^5^ in Beaverton, Orgeon, the authors (1) reviewed the BS literature, (2) discussed the state of the field with several current BS researchers and clinicians to identify gaps in the current research, and (3) compiled a needs assessment to identify topics of most importance to the community. We begin with excerpts from one patient's story in his own words.

## ZACH'S STORY

Hi, my name is Zach Rogers and I have Bloom's syndrome. I am 26 years old. I was born weighing 3 lbs, 15 oz and was full-term. I was in distress in the womb and was born by emergency C-section and taken by Life Flight to the local children's hospital where I spent 3½ weeks.

I was originally diagnosed with IUGR [intrauterine growth restriction] and the doctors tried to get me to “catch up.” I was placed on a feeding tube from age 2 months to 10 months. I began throwing up constantly and stopped eating by mouth. My parents decided that for me to get better we should remove the feeding tube altogether. I was checked daily for dehydration and reintroduced to eating foods. After a few days my appetite returned, and I never threw up again for 4 years (when I had the stomach flu). It was a lesson to all my doctors that I was being overfed and that I was not meant to “catch up.” I began to love food, and to this day I love all foods except Fig Newtons.

Throughout my first 5 years of life I was followed closely in the neonatal program and regularly met with a psychologist, audiologist, ophthalmologist, cardiologist, gastroenterologist, general pediatrician, and, of course, a geneticist. Although I saw a renowned geneticist, because my condition was so rare, he did not diagnose me with Bloom's syndrome. It was not until I saw a dermatologist at age 2 years old for red raised bumps on my face (telangiectasia) that it was suggested that I might have Bloom's syndrome. When the results came back positive it was a new journey for myself, my parents and family, and my doctors.

My childhood was normal and happy. I had friends and was like any other child except I was much smaller and needed to wear a hat and sunscreen in the summer time. I was able to do everything that my friends could do, I was just shorter than them. I never felt bullied by kids in my school. They were all very accepting and I had plenty of friends. I have always had people in public stare at me, but I do not let it get to me. The only problem I had in school and with friends is that the other kids liked to pick me up which I did not like. I got into the habit of digging my fingernails into their hands as they picked me up and that soon took care of that problem.

I had all my scheduled childhood immunizations in full doses with no side effects. I had the usual colds and flus but no more often than other kids my age. I did get a lot of ear infections and sinus infections as a child but had no serious illnesses.

When I was 11 years old, I was at the pediatrician's for a scout camp physical when a lump was discovered on my neck. My pediatrician said I could go to camp (1 week) but we had to watch it closely for changes and see a specialist immediately upon returning if the lymph node was still swollen. I had some weight loss but otherwise felt fine and the lump was not painful. After a CT scan it was determined that the lump was a harmless cyst but because of my syndrome they would remove it as a precaution. The surgeon recognized it as cancer as he was removing it, and I was soon diagnosed with large diffuse B cell non-Hodgkin's lymphoma. Although we knew my syndrome could cause cancer, it was unusual to get cancer at such a young age.

At the time of my diagnosis I was working with an oncologist who was familiar with Bloom's syndrome, Dr. Charles Keller. I will always be thankful that Dr. Keller was living in Utah at the time and knew enough about Bloom's syndrome to know that a normal chemotherapy course would be detrimental to my health and that I would need a very personalized treatment. I was treated under the CCG Protocol 5961 with the addition of rituximab and had very reduced levels of chemotherapy to avoid toxicity because of DNA instability associated with Bloom's syndrome.

My chemotherapy was planned for about 50% of normal doses for my weight. However, the levels were reduced even more and in the end my chemotherapy was personalized at one-eighth to one-fourth of the normal levels for my weight. Radiation therapy was out of the question because of my syndrome.

I underwent about 5 months of chemotherapy; in-patient for 1 week for chemotherapy, then went home for 2 weeks and watched closely for infection, etc. I had a very good attitude about my hospital visits and looked forward to ordering room service. I did lose all my hair with the reduced doses but did not end up in the hospital with an infection. I did have a severe reaction to the vincristine and was hospitalized for a weekend. About two-thirds into my treatment my weight had dropped too low, and I had to have a feeding tube. This was the hardest part of the treatment for me, and I remember feeling very angry about it. My therapy was a work in progress and adjustments were made as needed as we went along. After my treatment, I was followed with CT scans and watched closely. My lymphoma went into remission, and I have been cancer-free now for 15 years.

After my cancer treatment, it was discovered through routine follow-up blood work that my IgG levels were low. I was sent to an immunologist who ran more tests and discovered that I had no immunities to my childhood vaccinations, and although he was concerned about all my IgG levels, he was very concerned about my IgG2 level of 12. He gave me the Prevnar vaccine then checked my immune response 2 weeks later. There was no immune response. It was repeated twice with the same result. It was recommended that I receive IVIG therapy on a regular basis. There were a lot of conflicting opinions in the Bloom's syndrome community regarding the benefits of IVIG, and we struggled with the decision. I rarely got sick and there was no evidence, except on paper, that my immunities were low. My pediatrician concurred that I got sick less often than my siblings. It was assumed that my immunities had always been this low and were a symptom of the syndrome and not of the cancer treatment.

Eventually the decision was made that it was not worth the risk of having IgG levels that low, and I have received IVIG therapy (out-patient) in the hospital about every 8 weeks to this day. The therapy makes me feel better overall. I sleep better, I am not as fatigued during the day, and my allergies and stomach upset are improved. I can tell from my body when it is time to have IVIG, and it is personalized to when I decide to come in, usually about every 8–10 weeks. We have found that keeping the infusion slow reduces side effects such as kidney/back pain, and the nurses that administer it follow a treatment plan that is personalized for me that differs from the normal rate of flow for someone else my age or weight.

A few years after my cancer treatment there was an idea discussed among my doctors that we think about trying monthly Lupron injections to increase my height since growth hormones are not usually recommended for Bloom's syndrome patients. The Lupron injections would delay and lengthen puberty to allow more time to grow until my growth plates closed. I was told I was small even on the Bloom's syndrome curve, and I was open to the idea. We thought about it for a few months. My mom thought that my starting puberty might have caused my lymphoma, so she liked the idea of suppressing my hormones as I went through puberty as a protection against cancer. My doctors did not necessarily support this theory, but it made sense to my mom, so she agreed. I had monthly Lupron injections for about 5 years. My endocrinologist estimates that I gained about 4.5 in. in height by this personalized medical treatment. If I would not have had the injections I would most likely be that much shorter than I am now. I am now 4′8′′. To my knowledge no other Bloom's syndrome patient has tried this approach, but I did not have any negative effects and am happy to be a little taller.

Today I am happy and healthy and got married a month ago. I have a good job, a beautiful wife, and great doctors. I am living life to the fullest and trust that if I get cancer my doctors know enough about my syndrome and past treatment to treat me with my own personalized medicine. I have shared my cancer treatment protocol with others around the world and am happy to help anyone that I can. I am very open about my syndrome and treatments and feel like it is very important to share information within the Bloom's syndrome community. We connect with others by attending conferences and on social media.

## NEEDS AS IDENTIFIED BY FAMILIES AND PATIENTS AFFECTED BY BLOOM SYNDROME

As Zach's story highlighted, people with BS are faced with tremendous uncertainty about how to choose appropriate medical treatments for cancer and other consequences of BS. Important fundamental questions about the condition remain, and progress toward better care and a potential cure has been challenging. Herein, we report a road map consisting of three primary steps toward overcoming challenges to enable further progress.

### Road Map Step 1: Establishing Protocols for Cancer Surveillance and Treatment

Persons with BS and their families need an appropriate screening and surveillance program for cancer and to know how to treat cancers when they arise. In this area, two important advances have recently been made. First, the American Association of Cancer Researchers (AACR) recently published cancer screening and surveillance recommendations for persons with DNA repair disorders (Walsh et al. 2017). Second, building on this work, the director of the BSR is authoring an article (with substantial contributions from an expert committee) that will provide detailed health supervision guidelines primarily focused on cancer surveillance and screening but also addressing other related health issues associated with BS (C Cunniff, AR Djavid, S Carruba, et al., unpubl comm).

Dedicated efforts to track adherence to these surveillance protocols and their related outcomes will enable us determine their efficacy. Our hope is to identify protocols that will lead to lower mortality rates, as was seen in the Li–Fraumeni community when the Toronto protocol was followed (Villani et al. 2016), as well as to identify and eliminate those procedures that do not lead to better outcomes. Establishing standards and measuring effectiveness of protocols should also improve insurance reimbursement for this intensive procedure regimen.

Regarding the treatment of cancer in persons with BS once cancer has been diagnosed, a small amount of information exists, but even fewer data have been gathered. As referenced in Zach’s story above, typical cancer treatment regimens should be modified for BS cancers. Persons with BS are especially sensitive to DNA-damaging chemotherapy and radiation, and the risk of secondary cancers or myelodysplasia must be considered. Specific (most often unsuccessful) protocols have been published in isolated incidents (cf. Ma et al. 2001; Mizumoto et al. 2013), and modified protocols and resultant outcomes are only shared sporadically within the BS community. A vital step toward establishing treatment protocols is the collection of those that have been used previously and their outcomes. This information will help to form a basic understanding of the interplay between the genomics and cancer treatment for people with BS. Whereas this step will provide vital benefits by contributing to the development of evidence-based customized cancer treatments, there is also hope that this information will prove useful in the development of validated targets, as we discuss below. We recommend that the data collection described above be done as part of the BSR “Registry Data Project” described below.

### Road Map Step 2: Building the Components of a Successful Rare Disease Community

The small number of persons with any given rare disease is a challenge for understanding and curing the disease. However, recent advances in several areas (including communication through social media, new incentives for rare disease drug development, better understanding of rare diseases, and novel patient–researcher–industry relationships) lead us to believe that there is an opportunity today to make significant strides in the understanding of BS.

We reviewed successes in other rare disease communities (such as cystic fibrosis, muscular dystrophy, and Fanconi anemia [FA], among others) and identified components that are important for the BS community to develop and strengthen. Such components include a registry; a biorepository; strong, sustained patient participation; a collaborative research community; scientific leadership; disease models; a validated target; biopharma partners; novel clinical trial designs; and funding. Below, for each of those components, we outline the current state in the BS community and important goals to help strengthen our community.

#### Bloom's Syndrome Registry (BSR)

Since it was established in 1960, the BSR has operated intermittently, with limited funding and periods of closed enrollment. In 2014, a new director was appointed and is now on task to revitalize the organization. The BSR is integral to the research infrastructure needed for the BS community. To better position the BSR for tackling research questions of relevance to the community, we recommend initiation of a “Registry Data Project” with the following immediate goals: update of the website and outreach campaign; appointment of a patient advocacy advisory board; digitization of existing data, ensuring information management and access; and a schedule for ongoing data collection.

#### Biorepository

The BSR previously collected biological samples of persons with BS (including tumor samples), but little is known about the samples' condition and thus their availability for research. To our knowledge, tumor sequencing data (such as would facilitate the development of precision oncological treatments) are not available for any of these samples. In consultation with the advisory board, the BSR should reinstate and update this biorepository as the sole source of biological samples related to BS. Immediate priority samples include blood and fibroblast samples of registrants and tumor samples from registrants reporting cancers.

#### Strong Patient Participation

It is the authors’ assessment that families and persons with BS are cautiously willing to be partners with the research community, but some have been discouraged in the past by the lack of progress and information shared from the BSR and BS researchers. The dual research–patient advocacy model used for the RecQ conferences in 2008 (Ellis et al. 2008) and 2016,^6^ which we believe should continue, has helped build trust, but much work remains to build closer ties. In order to better explain the new guidelines on health supervision in plain language, and to encourage patient input back to clinicians and researchers, the authors of this paper are developing a handbook for families and persons with BS. An annual or biennial conference specifically focused on BS would further help build relationships within the patient and family population and with researchers and clinicians.

#### Collaborative Research Community

We could not identify any researchers working on translational research specifically investigating novel cancer treatments or curative treatments for BS. Individual oncologists of persons with BS have shown interest in learning from others in the field, but as of yet there is no “community of practice” for oncologists around treating BS cancers. As a community, we need to raise awareness about BS and highlight the importance of our unanswered research questions, not only for BS but also for cancer research and genetic research more generally. We should seek to engage researchers working on other genomic instability syndromes (e.g., FA, Li–Fraumeni, as well as the other RecQ disorders) to understand the potential for applying their work to BS. As a community, we should recognize and promote those researchers that have taken a leadership role and continue to raise awareness about BS to help establish more attention from the funding agencies.

#### Disease Models

One challenge to better understanding BS is the lack of robust animal models that faithfully reproduce the pathological processes and phenotypes seen in humans. Mice with loss of function of *Mus* (*Blm* analog in the house mouse) die in the embryonic phase; yet there does exist a hypomorphic *Blm* mutation mouse that may be promising for preclinical research (Cunniff et al. 2017). Pigs are known to be more analogous to humans in many ways, especially for their use in cancer research (Watson et al. 2016) but are more expensive to procure and maintain, and we are unaware of any attempts to create a porcine model of BS or BS cancers. Zebrafish have been noted for their suitability in studying DNA damage and repair (Pei and Strauss 2013), and a zebrafish model for BS does exist yet lacks phenotype data (Cunniff et al. 2017). Cell lines for BS exist and are available from cell repositories, including the Coriell Institute for Medical Research,^7^ but not all BS mutations recognized in the BSR are represented in these cell lines. Although there were 64 different mutations noted in the BSR as of 2007 (German et al. 2007), Coriell provides only 12 such mutations. As a community, we need to fully catalog disease models available, continue to grow the pool of suitable models, and help make these available to researchers. As the collection of BS tumors grows in our biorepository, research will also be positioned to benefit from patient-derived xenografts (PDX), either in mice or zebrafish (Fior et al. 2017) or other systems, to study cancers of BS and to develop treatments.

#### Validated Target

As of right now, we do not have a validated target for developing a therapy for BS nor for precision treatment of BS cancers. Through a series of experiments throughout the 1970s, 1980s, and 1990s, the *BLM* gene was identified and was mapped to Chromosome 15 (Cunniff et al. 2017). Although much research continues, a complete understanding of the role of the associated BLM protein and its interaction with other proteins is lacking. Many different mutations are known to be pathogenic (German et al. 2007), and different mutations may have different “druggability” depending on the approach to treatment pursued (e.g., a nonsense mutation may be amenable to exon skipping or readthrough, whereas other mutations may require introduction of the functional gene). With respect to targets for cancer treatment, we think the potential for the development of precision BS cancer treatments using a synthetic lethality concept is particularly enticing (O'Neil et al. 2017).

As part of the toolkit for developing validated targets for curing BS, several assays exist for measuring *Blm* production. Standard techniques such as western blot analysis and immunofluorescence are used in the literature. Some specific assays used for BS are the measurement of the level of sister chromatid exchange (SCE) levels, which are elevated significantly (∼10×) in BS cells (Chaganti et al. 1974). SCE levels have been shown to correlate with the level of Blm expressed (Cunniff et al. 2017), and thus serve as a useful endpoint.

#### Clinical Trial Designs

The Food and Drug Administration (FDA) in the United States has shown a willingness to work with patient communities in rare diseases, recognizing that those communities are often themselves the “experts” and should weigh in on the tradeoffs on risk and uncertainty that might be acceptable. The FDA has a designated Rare Diseases Program and has provided guidance on common issues in rare disease drug development.^8^ The European Medicines Agency (EMA) has similarly showed an understanding of the challenges in rare diseases, and the EU has funded research on small population clinical trials.^9^ As work progresses, our community will need to involve the FDA and the EMA early in discussing clinical trial design. Natural histories collected as part of the BSR, as well as revitalizing the BSR ongoing data collection, will be vitally important to have.

#### Funding

There is almost no dedicated, sustained funding source for BS research. The BSR is supported in part by a small grant, but needs additional funding. Each of the above components will require funding, and our community will need to help identify additional sources of funding, either through federal or foundation funding, or through capital campaigns by the BS community.

### Road Map Step 3: Identifying Research Questions of Most Interest to the BS Community

The above components of a robust rare disease community infrastructure must be realized in order to address research questions and spur development of treatments or therapies. Our needs assessment identified research questions around three major themes.

1. More effective, less toxic cancer treatments
  - Precision Oncology in Bloom syndrome. Are there specific genetic mutations or other signatures unique to BS tumors that would make these tumors more susceptible to certain treatments, analogous to the poly(ADP) ribose polymerase (PARP) inhibitors used for BRCA-mutated tumors (Lord and Ashworth 2016)? Are tumors in BS inherently more responsive to chemotherapy because of their reduced efficiency at DNA repair, such as microsatellite instability (MSI)-high tumors seen in Lynch syndrome (Kawakami et al. 2016)? Can an understanding of these features be tied back to more strongly evidence-based BS cancer treatment protocols?
  - Immuno-oncology. Unleashing the human immune system to fight cancers, either through checkpoint inhibitors or by genetically engineering T cells (including CAR-T) to better target cancer without the toxic side effects of chemotherapy or radiation is being hailed as the fifth pillar of cancer care.^10^ Such treatment could be tremendously important for BS cancer patients, who are more susceptible to secondary neoplasms resulting from chemo or radiation. However, the immune system is affected in BS (Babbe et al. 2007), and it seems reasonable to suspect that genetic engineering may face additional challenges in BS cells due to those cells’ impaired DNA repair mechanisms. Would checkpoint inhibitors work in persons with BS? Can BS T cells be reprogrammed to fight cancer? If so, can such reprogramming be done to not only modify the T-cell receptors, but also to repair the BS gene in T cells to make these cells behave more like healthy T cells?
2. Fundamental unknowns around Bloom syndrome clinical features
  - Growth. The short stature observed in persons with BS is not fully understood. Growth hormone production and secretion are normal (Diaz et al. 2006), yet growth hormone treatment has shown to increase linear growth (Renes et al. 2013). What is the cause of the short stature—a cell proliferation deficit, increased cell death, or something else? Metabolic studies may be in order to answer these questions.
  - Rash. There appears to be insufficient evidence as to whether the rash in BS (Giordano et al. 2016) is actually caused by UV exposure, although persons with BS report the onset or exacerbation of the rash with sun exposure, BS cells exposed to UV in vitro do not show damage (Cunniff et al. 2017). A better animal model of BS may elucidate this in more detail. Furthermore, there have been no reported recommended treatments for the rash.
  - Immunodeficiency. What is the cause (or mechanism) of immunodeficiency in BS, and what is the interplay between this and immunotherapy for cancer treatment?
  - Fertility. It has been reported that females with BS are subfertile and males with BS are infertile. However, up to date studies have not been performed that definitively describe the cause behind this (for review, see Cunniff et al. 2017, which primarily points to a study done in 1987), and there is at least one report of a male with BS fathering two children (Ben Salah et al. 2014). What is the mechanism by which BS causes infertility? Is it possible that this varies by *BLM* mutation?
3. Restoring (or replacing) BLM in vivo
  - Nonsense suppression. Mutations in *BLM* leading to BS can come in many forms (German et al. 2007), but several of the mutations are nonsense mutations or premature termination codons (PTCs) in which the translation of the gene into protein is stopped mid-translation, leading to a nonfunctional protein. A therapeutic avenue being explored for other genetic conditions associated with PTCs is small molecule readthrough or nonsense suppression (Bordeira-Carriço et al. 2012; Lee and Dougherty 2012); this has been studied preclinically for many different conditions, including another RecQ disorder, Werner syndrome (Agrelo et al. 2015). Advances in this area have been limited by the most commonly used small molecules’ toxicity, but recently it was discovered that the relevant active component of a common readthrough molecule, gentamicin, can be used at higher levels with minimal toxicity (Baradaran-Heravi et al. 2017). Do existing readthrough compounds have any effect on BLM cells containing PTC mutations? If so, is the nonsense suppression sufficient to restore BLM and reduce SCEs to a therapeutic level? Interestingly, and related to the questions around the BS rash, there have been reports of these readthrough molecules being applied topically for other conditions that have nonsense mutation-associated rashes (Kellermayer et al. 2006; Kuschal et al. 2013).
  - Exon skipping. Another therapy being developed for other rare genetic disorders is exon skipping, in which the mutated exon is “skipped over” by using an antisense oligonucleotide.^11^ For BS mutations outside of the helicase domain, this may be worth exploring. Would BS mutations be amenable to exon skipping?
  - Compensation for BLM. The human body contains five RecQ helicases which are each involved in various aspects of DNA replication and repair. In the absence of BLM, are other RecQ helicases called to pitch in? It has been reported that RECQL5 may help suppress SCEs when BLM function is impaired (Wang et al. 2003; Croteau et al. 2014). If so, could it be up-regulated to compensate for the lack of BLM? It has been demonstrated in cells that using RecG from *Escherichia coli* can reduce SCE levels (Killen et al. 2012)—is this translatable to a therapy?
  - Gene therapy or gene editing. The development of CRISPR-type technology has revolutionized the prospects for gene editing (Porteus 2016). Researchers have shown the ability to correct the FA gene in patient-derived induced pluripotent cells (Osborn et al. 2015), which is notable given FA is also a DNA repair-deficient disorder and may have some of the challenges expected for gene editing in BS cells. Could a similar approach be taken to correct the BLM gene in BS cells? If so, could ex vivo correction and reintroduction (minus traditional ablative preparation) of stem cells be a viable approach to avoid leukemias in BS?

## CONCLUSION

Other underserved patient populations are finding innovative academic, foundation, and industry arrangements to improve care and therapies for their communities (Ramsey et al. 2017) and have shown the value of standard cancer screening and treatment protocols (Villani et al. 2016). Although we in the Bloom syndrome community face additional challenges given the rarity of the condition, we are optimistic that by following the above road map, we may make great strides in improving care for persons with BS and progressing toward a cure.

### Competing Interest Statement

The authors have declared no competing interest.

## ADDITIONAL INFORMATION

### Acknowledgments

We thank the Children's Cancer Therapy Development Institute and its Scientific Director, Dr. Charles Keller, for all of their work in hosting the 2017 Pediatric Cancer Nanocourse. We also thank the Bloom Syndrome speakers at the Nanocourse, including Dr. Chris Cunniff, Dr. Nathan Ellis, Dr. Mark Osborn, and Dr. Sharon Plon, for participating.

## Footnotes

4 There has been inconsistent use in the literature regarding the possessive (Bloom's) versus the nonpossessive (Bloom) form of BS. Herein, we choose to use the nonpossessive form but recognize the possessive form used in the names of existing organizations.

5 http://www.cc-tdi.org/ (accessed September 8, 2017).

6 https://www.fredhutch.org/en/events/recq2016-partner-in-progress.html (accessed September 8, 2017).

7 https://catalog.coriell.org/Search?q=%22BLM%22 (accessed September 8, 2017)

8 https://www.fda.gov/downloads/Drugs/GuidanceComplianceRegulatoryInformation/Guidances/UCM458485.pdf (accessed September 8, 2017).

9 http://www.ema.europa.eu/ema/index.jsp?curl=pages/news_and_events/events/2017/02/event_detail_001393.jsp&mid=WC0b01ac058004d5c3 (accessed September 8, 2017).

10 http://www.ascopost.com/issues/may-25-2016/how-immunotherapy-is-revolutionizing-cancer-care/ (posted May 25, 2016; accessed September 8, 2017).

11 http://blogs.plos.org/dnascience/2014/02/20/exon-skipping-borrowing-nature-treat-rare-genetic-diseases/ (posted February 20, 2014; accessed September 8, 2017).
